# Application of ATC/DDD methodology to eveluate of antibiotic use in a general hospital in Turkey

**DOI:** 10.1186/1476-0711-12-23

**Published:** 2013-09-03

**Authors:** Hamdi Sözen, Ibak Gönen, Ayse Sözen, Ali Kutlucan, Serdar Kalemci, Murat Sahan

**Affiliations:** 1 Medical Faculty, Department of Infectious Diseases and Clinical Microbiology, Mugla Sıtkı Kocman University, Mugla, Turkey; 2 Medical Faculty, Department of Infectious Diseases and Clinical Microbiology, Suleyman Demirel University, Isparta, Turkey; 3 Department of Neurology, Mugla General Hospital, Mugla, Turkey; 4 Medical Faculty, Department of Internal Medicine, Duzce University, Duzce, Turkey; 5 Medical Faculty, Department of Chest Diseases, Mugla Sıtkı Kocman University, Mugla, Turkey; 6 Medical Faculty, Department of Otolaryngology Head and Neck Surgery Mugla, Mugla Sıtkı Kocman University, Mugla, Turkey

**Keywords:** Antibiotic, ATC/DDD index, Appropriate use of antibiotics

## Abstract

**Background:**

The aim of this study is to evaluate in-house antibiotic use in a state hospital in Turkey with its cost, using the ATC/DDD index, which is an accepted standard method.

**Methods:**

This study was performed as a point prevalence study in a state hospital with 372 beds. All in-house patients using antibiotics on July 19, 2011 were included in the study. Indications for antibiotic use and information about the patients were recorded on special forms. Antibiotic use and cost analysis were evaluated using the ATC/DDD index, which is also suggested by the WHO to be used in similar studies.

**Findings:**

147 patients out of 308 patients who were in-house were identified to use antibiotics with appropriate indications for prophylaxis or treatment in 61% of the patients. The rate of appropriate antibiotic use was identified to be in 78%, while this rate was 38.9% in surgical clinics. The daily cost of the antibiotics consumed on the date of the study was calculated as 4104.79 TL (=2476.80 USD).

**Discussion:**

The rate of inappropriate use of antibiotics seems to be high in our hospital. This will result in both increased costs and also increased nosocomial infection rates with resistant species. Infectious disease specialists should take more active roles in the in-house antibiotic use, hospitals should prepare and implement their own principles of antibiotic use, and microbiology laboratories should be used more effectively. These measures would decrease the conspicuous shortcomings in the antibiotic use.

## Introduction

As the anti infective agents are most frequently used group of drugs in our country, also doctors most frequently error when chosing them [1,2]. Improper use of antibiotics often lead to many problems such as; development of resistance to antibiotics, negative effects on the ecological balance, side effects on patients, triggering of superinfections and increase in treatment costs [3-5]. The most important feature that separates antibiotics from other drugs is that their improper use does not only negatively affect patients but also has negative impact on the hospital microbial environment. As a result, while infections caused by resistant microorganisms gradually increase, antimicrobial options used for treating them rapidly diminish [6,7]. In this case, the accurate determination of antibiotic therapy will prevent the use of incorrect and inappropriate antibiotics [8].

Unnecessary use of antibiotics is a major worldwide problem. Despite of detailed research on trends of antibiotic use in and out of the hospitals in many countries, no sufficient data is available for our country [9].

Because each antibiotic has different unit dose of daily administration, a specific standardized method should be used in the evaluation of in-hospital antibiotic use. Of the current standard methods, the method suggested by the World Health Organization (WHO) is the prominent. The ATC/DDD index is developed and intervaly updated by the WHO. The WHO Collaborating Centre for Drugs Statistics Methodology has standardized this with the ATC/DDD (Anatomical Therapeutic Chemical/Defined Daily Dose) index [10]. DDD 100 bed-days has been used internationally in the comparison of in-hospital and outpatient antibiotic use, and such data have been used to compare and to throw light on the national level of antibiotic use and resistance relationship [11]. Calculating antibiotic use intensity with the ATC/DDD index is independent from the price and box dimensions, and the daily dose for every antibiotic expresses the same DDD. By using this method, a comparison of antibiotic use can be made not only in clinics and hospitals but at the same time among countries. However, because it is based on adults only, low or high dose applications due to chronic renal and liver failure can change the sum. Generally, the ATC/DDD index is a universal parameter used in the evaluation of antibiotic use [12-17].

The aim of this study was to evaluate the use and cost of antibiotics in a Turkish state hospital by a point prevalence research with the ATC/DDD index which is accepted as a standard.

## Methods

Isparta State Hospital is a secondary healthcare facility with 372 bed capacity and 4 different intensive care units with 21 bed capacity.

A research of modified point prevalence research was carried out in order to evaluate the frequency and appropriate use of antibiotics in our hospital. The data of this study were obtained from 7 departments of internal diseases (internal medicine, neurology, chest diseases, cardiology, dermatology, physical therapy and rehabilitation(PRT), infectious diseases), 8 departments of surgical diseases (neurosurgery, Ear, Nose, and Throat/Head and Neck Surgery(ENT), cardiovasculer surgery(CVS), thoracic surgery, general surgery, orthopedics, urology and plastic surgery) and 4 intensive care units (anesthesia, internal medicine, neurology, chest diseases). Data concerning antibiotic use in the hospital were collected on the 19th of July 2011 by an infectious diseases specialist and all patients receiving antibiotics were included to the study. Data were collected from patient charts using a standard form; patients personal information, underlying disease, the name of the antibiotic in use, dosage, usage, duration, reason of antibiotic use (prophylaxis, empyrical, microbiologically prooven infection) and microbiological test results were recorded on the form.

Th existence of an infectious disease was detected by evaluating patient’s clinical complaints, physical examination findings and laboratory findings all together. In diagnosing nosocomial infections, CDC (The Centers for Disease Control) criteria and NNIS (National Nosocomial Infections Surveillance System) methodology were used [18]. The antimicrobial suitability was evaluated according to the criteria described by Kunin and Jones and The Sanford Guide to Antimicrobial Therapy [19-21].

Surgical prophylaxis was evaluated taking into account the drug dose, administration way, duration of prophylaxis and international guidelines [22].

Patients using antibiotics were evaluated according to suitable indication, dose, administration way and criteria for adequate antibiotic. When evaluated by the infectious diseases specialist, patients matching all criteria were accepted as “suitable”, and in the absence of even one criteria the patient was “non-suitable”.

The DDD is the assumed average maintenance dose per day for a drug used for its main indication in adults. Only drugs with an ATC code can have DDD values. The DDD value in grams of every drug is defined by the WHO and is periodically updated. DDD values of every antibiotic is calculated separately [10].

Defined Daily Doses (DDDs) are calculated separately for every antibiotic, the average maintenance dose for an adult weighing 70 kg is prepared in main indications and the active substance should be taken as grams (or I.U.).

DDDs = Number of boxes x number of tablets in the box or number of vials x tablets in grams or the weigh of the vial / the DDD value of the antibiotic in grams.

In this calculation method, the form used for in-bed patients is the ratio of the total DDD per 100-bed-days. This index is called antimicrobial consumption index (ACI).

ACI=DDD/bed−days×100

Also, the antibiotic consumption index of a country or geographical area at a certain period of time is calculated by DDD per 1000 people.

The number obtained is the antibiotic consumption index of that hospital/clinic or population.

In this study, DDDs of anti infective agents are listed for systemic use according to ATC/DDD 2010 Index.

The total cost of every antibiotic used in the hospital was calculated according to the price list of the General Directorate of Pharmaceuticals of The Republic of Turkey on the 19th of July 2011. Afterwards, the cost was converted to USD according to the exchange rates of the Central Bank of The Republic of Turkey on the 19th of July 2011 (1 USD = 1,6573 TL).

## Results

The mean age of the 308 patients was 56.7 + 18.2. Of the patients included to the study, 161 were male (52.3%), and 147 were female (47.7%). Of the 308 patients, 147 (47%) received antibiotics for any reason. Of the 147 patients, 47 (32%) received antibiotics for surgical antimicrobial prophylaxis, 92 (62.6%) empirical antimicrobial therapy and only 8 patients (5.4%) received antibiotics based on microbiological data.

When evaluating the 147 patients using antibiotics, 61.9% were evaluated as “appropriate” and the rate in the clinics of internal diseases was 76.6%, in the surgical clinics 38.9%, in the intensive care units 81.8%. According to the microbiological data, of the 8 patients receiving antibiotics 5 are intensive care patients, and while 45.5% of the patients from the intensive care units receive antibiotics according to microbiological data, in the departments of internal and surgical diseases this is only 2.6% and 1.7% respectively (Table 1).

**Table 1:** Antibiotic use variables in medical units

	**Number of patients**	**Antibiotic usage rate (%)**	**Rate of appropriate antibiotic use (%)**	**Evidence based antibiotic use rate (%)**
Internal med. units	181	42.5	76.6	2.6
(Acute exacerbation of COPD)	(49)	(89.8)	(72.7)	(4.5)
Surgical units	113	52.2	38.9	1.7
(Surgical prophylaxis)	(101)	(46.5)	(27.7)	(0)
Intensive care units	14	78.6	81.8	45.5
Total	308	47.7	61.9	5.4

The rate of antibiotic use in the departments for internal diseases was 42.5%, in the surgical departments 52.2% and in the intensive care units 78.6% (Table 1). While the highest rate of antibiotic use in the departments for internal diseases was in the department for pulmonary disease 89.8%, in infectious diseases 75% and in dermatology 66.7%, the lowest rate was found to be in the department for internal medicine with 7.1% and the neurology clinic with 4.8%. In the surgical departments; chest surgery (70%), plastic surgery (66.7%), brain surgery and CVS (60%) had the highest rates of antibiotic use while, urology and ENT (33.3%) had the lowest rates of antibiotic use (Table 2).

**Table 2:** Antibiotic usage rates of medical units

**Internal medicine units**	**The number of patients to which antibiotics were administered/ number of patients**	**%**	**Surgical units**	**The number of patients to whom antibiotics were administered/ number of patients**	**%**
Internal medicine	3/42	7.1	General surgery	19/35	54.3
Neurology	1/21	4.8	Orthopedics	12/26	46.2
Cardiology	4/19	21.1	Neurosurgery	6/10	60
PTR	2/20	10	ENT	3/9	33.3
Dermatology	8/12	66.7	Plastic surgery	6/9	66.7
Infectious diseases	6/8	75	CVS	3/5	60
Pulmonary dis.	53/59	89.8	Chest surgery	7/10	70
Total	77/181	42.5	Urology	3/9	33.3
Intensive care unit	11/14	78.6	Total	59/113	52.2

*PTR* Physical Medicine and Rehabilitation, *CVS* Cardiovasculer Surgery, *ENT* Ear, Nose, and Throat/Head and Neck Surgery.

The mean duration of surgical prophylaxis was 4.74 days (minimum 1, maximum 17 days). Duration of surgical prophylaxis according to the clinics was; orthopedics 9.5, thoracic surgery 6.8, general surgery 2.1, urology 1.4, ENT 1.3 days, respectively. The only service where third generation cephalosporins were used in surgical prophylaxis was thoracic surgery.

In our study, cephalosporins were found to be the most frequently used antimicrobials among all antibiotics, with a rate of 57%. Among the cephalosporins, 3rd generation cephalosporins (including beta lactamase inhibitors) constituted 21% of all antibiotics used. First generation cephalosporins were most frequently used with a rate of 20.8%. Fluoroquinolones and penicillins (including beta lactamase inhibitors) were the groups prefered after cephalosporins. In the departments for internal diseases most frequently preferd antibiotics were 2nd generation cephalosporins and fluoroquinolones, in the surgical departments 1st generation cephalosporins, and in the intensive care units 3rd generation cephalosporins (including beta lactamase inhibitors) and carbapenems(Table 3).

**Table 3:** Antibiotics usage rates of medical units

**Antibiotics**	**ATC code**	**Internal medicine units**		**Surgical units**		**Intensive care units**		**Total**		**Total rate**
		**DDDs**	**ACI**	**DDDs**	**ACI**	**DDDs**	**ACI**	**DDDs**	**ACI**	**%**
Penicillins	J01CR	16.9	9.3	0	0	0	0	16.9	5.5	10
First generation cephalosporins	J01DB	3.3	1.8	30.7	27.2	1.3	9.3	35.3	11.5	20.8
Second generation cephalosporins	J01DC	23	12.7	3	2.7	0	0	26	8.4	15.2
Third generation cephalosporins	J01DD	19.8	10.9	10	8.8	6	42.9	35.8	11.6	21
Carbapenems	J01DH	2.1	1.2	1	0.9	2	14.3	5.1	1.7	3.1
Aminoglycosides	J01GB	0.8	0.4	6.7	5.9	0	0	7.5	2.4	4.4
Imidazoles	J01XD	0	0	3	2.7	0	0	3	0.9	1.7
Glycopeptides	J01XA	3	1.7	0	0	1	7.1	4	1.3	2.4
Fluoroquinolones	J01MA	22.9	12.7	1	0.9	1	7.1	24.9	8.1	14.7
Others		10.3	5.7	0	0	1	7.1	11.3	3.7	6.7
Total		102.1	56.4	55.4	49.1	12.3	87.8	169.8	55.1	100

Total days of use was calculated to be 55.1 DDD/100 bed-days, 56.4 in the departments of internal diseases, 49.1 in the surgical departments, while in the intensive care units was found to be 87.8 DDD/100 bed-days(Table 4).

**Table 4:** Cost per infected patient and ACI rates of medical units

	**Cost per infected patient (TL)**	**Cost per infected patient ($)**	**ACI**
Internal med. units	36.27	21.98	56.4
(Acute exacerbation of COPD)	20.48	12.36	106.1
Surgical units	12.98	7.86	49.1
(Surgical prophylaxis)	5.88	3.55	41.4
Intensive care units	49.64	29.95	87.8
Total	27.92	16.89	55.1

The study was carried out on 19 July 2011, the total amount of all antibiotics were used in-hospital and the total cost of antibiotics used was calculated according to the price list of the General Directorate of Pharmaceuticals of the Republic of Turkey on the same day. On the day of the research the daily cost of the antibiotics used in our hospital was 4104.79 TL (=2476.80 USD). The cost per infected patient was 27.92 TL (=16.89 USD). In the intensive care unit daily antibiotic costs were 546.08 TL (=329 USD), per infected paient daily costs were 49.64 TL (=29.95 USD). In the departments of internal diseases daily antibiotic costs were 2793.08 TL (1685 USD), per infected patient being 36.27 TL (=21.89 USD). Among the departments of internal diseases, the department for chest diseases had the most frequent antibiotic use and te highest number of in-bed patients. The daily antibiotic costs of the department for chest diseases was 1126.45 TL (679.69 USD), of which 20.48 TL (=12.36 USD) per patient/day. Total antibiotic costs per day in the surgical departments were found to be 765.63 TL (= 463.98 USD), of which per infected patient 12.98 TL (=7.86 USD)(Table 4).

## Discussion

The use of antimicrobial agent does not only diverse between countries, but also diverse between the hospitals of a same country. These differencies can be correlated with hospital and patient features, antibiotic policies of the hospitals, physicians preferences and with the differencies in the educational and health systems [14].

Antimicrobial agents are the most frequently used drugs in Turkey and they constitue 22% of all drugs used [2].

In our study, the rate of antibiotics used was found to be 47.7%. In similar studies performed in our country the in-hospital rate of antibiotic use ranges between 45.6% and 61%, coinciding with the rates we detected [8,23,24]. In the Northern European countries the in-hospital rates of antibiotic use are lower than in our country, ranging between 16.6% and 25% [25,26].

In this study the proper antibiotic rate of use was found to be 61.9%. While in the departments for internal diseases this rate was 76.6%, in the surgical departments and in the intensive care units the rates were 38.9% and 81.8% respectively. While 32% of the patients received antibiotics for surgical prophylaxis, the use of antibiotics for acute exacerbation of COPD (29.9%) and penumonia (14.3%) draws our attention. High rate of antibiotic use is acceptable because one of the major pharmacologic treatment used in acute exacerbation of chronic obstructive pulmonary disease(COPD) are antibiotics [27].

When we evaluated the duration of surgical prophylaxis as a whole, the proper antibiotic choice, administration time and way were found to be only 27.7% appropriate. The major reason of the low proper antibiotic use rate in the surgical departments compared to other departments were the errors in the use of surgical prophylaxis. While the antibiotic for surgical prophylaxis was largely properly chosen, what was improper was the duration of prophylaxis. The mean duration of surgical prophylaxis was found to be 4.74 days. In surgical units, where surgical prostheses are implanted, surgical prophylaxis has been observed to be more inappropriate. Surgical prophylaxis was most frequently used in the orthopedics service. Average duration of surgical prophylaxis was 9.5 days and all of the patients were using more than one drug combination containing antibiotics. Urology and ENT were services that implement the most appropriate surgical prophylaxis. In similar studies, no errors were seen in the agent used in the prophylaxis, while the unnecessary extension of therapy was the main problem [28,29]. The proper antibiotic use in the intensive care units compared to the internal diseases and surgical departments was the use of antibiotic according to ‘evidence based’ (45.5%), and more regularly obtained consultations from the department for infectious diseases. The antibiotic use according to ‘evidence based’ in our hospital consist only 5.4% of the total antibiotic use and it is similar to other studies performed in our country [30,31]. In order to improve the improper use of antibiotics, eductional activities should be performed periodically, policies of current antibiotic use should be formed by the infection committees of the hospitals and the clinical practices should be controlled.

According to our study cephalosporins constitute 57% of the antimicrobials used in our hospital. Cephalosporins are followed by fluoroquinolones with 14.7% and penicillins with 10% of use. Of the cephalosporins used, 21% were 3rd generation, 20.8% 1st generation and 15.2% 2nd generation, which ranks on the first 3 places of the total drugs used. According to the ARPAC (Antibiotic Resistance, Prevention and Control) project, all hospitals most frequently used penicillins, followed by non-penicillin beta lactams and fluoroquinolones [32]. Antibiotic utilization rate in Turkey was found to be higher compared to the European countries. Especially the use of cephalosporins, penicillins and fluoroquinolones is much higher than in the European countries [33,34]. The frequent use of cephalosporins and fluoroquinolones lead to the emergence of resistant microorganisms, thus problems such as the emergence of resistant pathogenes in our area would be an inevitable consequence [35].

The ACI in our hospital was found to be 55.1 DDD/100 bed-days. In the intensive care units the ACI was 87.8 DDD/100 bed-days, in the departments for internal diseases 56.4 and in the surgical departments 49.1 DDD/100 bed-days. Eventhough the proper antibiotic use in the surgical departments was found to be lower than the department for internal diseases and intensive care units, the ACI value was lower. According to Akalın et al.’s study [23] in a university hospital, the ACI in 2009 was found to be 64.5 DDD/100 bed-days while in 2010 70.5 DDD/100 bed-days. These values are higher than the values in our study. This fact can be linked to the fact that in tertiary hospitals clinically more complicated and seriously ill patients are being treated compared to secondary hospitals. In a study by Vaccheri et al. [36] conducted in an university hospital in Italy it was shown that, the amount of antibiotics used, rised from 64.9 DDD/100 bed-day in 2002 to 76.7 DDD/100 bed-days in 2004. In the ARPAC Project conducted in 130 European hospitals, the antibiotic use was found to be 792 + 147 DDD/1000 bed-days. According to this large scale study, the antibiotic use in our country is parallel with southern and western Europe but higher than northern, middle and eastern European countries [32].

If we keep in mind that antibiotics are the most frequently used drugs in the hospitals, than we would know that they constitute an important part of the total drug expenditure. In our hospital the defined cost per infected patient per day was 16.85 USD. As expected, the antibiotic use in the intensive care units for infected patients was higher than in other clinics. Antibiotic use in the surgical departments was found to be 7.86 USD per infected patient per day, which is lower than other departments. Despite of the unnecessary extension of surgical prophylaxis, the reason of this is the choice of less expensive 1st generation cephalosporins. However, as the improper use of antibiotics is still high, the in-hospital antibiotic expenditure can be reduced even more. Reducing improper use of antibiotics would prevent the incidence of life-threatening serious infections, also preventing long hospital stays and higher health expenditures. The department for pulmonary diseases draws attention with the highest number of patients and also with the highest number of patients using antibiotics. The department for pulmonary diseases uses 27.5% of the daily antibiotic costs. Vast majority of the patients are hospitalized in this department due to diseases caused by smoking. Smoking can be considered as one of the factors that increases the use of antibiotics in hospitals.

Our study, carried out with point-prevalence method has certain limitations such as being single-centered, not having resistance ratios calculated and involving only one day of the year. The study was carried out in the summer period. Especially pneumonia and acute exacerbation of COPD are diseases seen more oftenly in the winter, and clinically more complicated diseases are less encountered in the summer period. However, by applying a method inexpensive and easy to implement as the point-prevalence, we can say that we gained detailed knowledge of the antimicrobial use in our hospital.

As a result, the use of ATC/DDD system in hospitals would provide internationally valid data in the evaluation of antimicrobial use. We believe that, more efficient utilization of infectious diseases experts in the use of antibiotics in hospitals, creating of guides for antibiotic use specific to every hospital and more efficient use of the microbiological laboratories may be of benefit in the resolving of existing problems.

## Consent

Written informed consent was obtained from the patient’s for the publication of this report.

## Competing interests

The authors have no competing interests to declare.

## Authors’ contributions

HS involved in designing of the project, collection of data, data analysis and interpretation, and write up of the manuscript. IG designed the study, and prepared the manuscript for publication. Other Authors: Collection of data. All authors read and approved the final manuscript.
